# Evaluating the Impact of Two Generalist Predators on the Leafhopper *Erasmoneura vulnerata* Population Density

**DOI:** 10.3390/insects12040321

**Published:** 2021-04-06

**Authors:** Stefan Cristian Prazaru, Giulia Zanettin, Alberto Pozzebon, Paola Tirello, Francesco Toffoletto, Davide Scaccini, Carlo Duso

**Affiliations:** Department of Agronomy, Food, Natural Resources, Animals and Environment, University of Padova, viale dell’ Università 16, 35020 Legnaro, Italy; giuliazanettin22@gmail.com (G.Z.); paola.tirello@unipd.it (P.T.); francesco.toffoletto@studenti.unipd.it (F.T.); davide.scaccini@phd.unipd.it (D.S.)

**Keywords:** augmentative biological control, invasive pest, grapevine, *Chrysoperla carnea*, *Orius majusculus*, predation

## Abstract

**Simple Summary:**

*Erasmoneura vulnerata*, a grapevine leafhopper native to North America, was detected in Europe (North-eastern Italy) in the early 2000s. Although it is considered a minor pest in its native range, outbreaks of this species have been reported in North-eastern Italy. In this study, we investigated the potential of two generalist predators, i.e., *Chrysoperla carnea* and *Orius* *majusculus*, in suppressing *E. vulnerata* in laboratory and semi-field experiments. Both species significantly reduced nymph numbers in laboratory and semi-field conditions. For this reason, field trials were performed. Predator releases in vineyards reduced *E. vulnerata* abundance by about 30%. Since naturally occurring *E. vulnerata* antagonists exert a moderate impact and the effectiveness of natural insecticides is limited, the augmentative release of generalist predators can be considered a complementary tool in controlling *E. vulnerata* populations in vineyards, particularly in organic farms.

**Abstract:**

Outbreaks of the Nearctic leafhopper *Erasmoneura vulnerata* represent a threat to vinegrowers in Southern Europe, in particular in North-eastern Italy. The pest outbreaks are frequent in organic vineyards because insecticides labeled for organic viticulture show limited effectiveness towards leafhoppers. On the other hand, the naturally occurring predators and parasitoids of *E. vulnerata* in vineyards are often not able to keep leafhopper densities at acceptable levels for vine-growers. In this study, we evaluated the potential of two generalist, commercially available predators, *Chrysoperla carnea* and *Orius majusculus*, in suppressing *E. vulnerata*. Laboratory and semi-field experiments were carried out to evaluate both species’ predation capacity on *E. vulnerata* nymphs. The experiments were conducted on grapevine leaves inside Petri dishes (laboratory) and on potted and caged grapevines (semi-field); in both experiments, the leaves or potted plants were infested with *E. vulnerata* nymphs prior to predator releases. Both predator species exhibited a remarkable voracity and significantly reduced leafhopper densities in laboratory and semi-field experiments. Therefore, field studies were carried out over two growing seasons in two vineyards. We released 4 *O. majusculus* adults and 30 *C. carnea* larvae per m^2^ of canopy. Predator releases in vineyards reduced leafhopper densities by about 30% compared to the control plots. Results obtained in this study showed that the two predators have a potential to suppress the pest density, but more research is required to define appropriate predator–prey release ratios and release timing. Studies on intraguild interactions and competition with naturally occurring predators are also suggested.

## 1. Introduction

*Erasmoneura vulnerata* (Fitch) (Hemiptera: Cicadellidae) is native to North America where it is reported as a minor pest of grapevines compared to other leafhopper species [1]. This pest was detected in North-eastern Italy (first record for Europe) in 2004 [2] but no population outbreaks were observed in the newly invaded areas until 2016 [3]. Since then, the pest status of *E. vulnerata* has increased in North-eastern Italy, particularly in the Veneto region. Meanwhile, *E. vulnerata* spread first to Slovenia [4], then to North-western Italy and Switzerland [5]. Studies carried out in North-eastern Italy showed that *E. vulnerata* completes three generations per year, and overwintered adults have a remarkable edge effect in vineyard colonization [6]. Adults and nymphs feed on the mesophyll causing leaf discolorations and premature leaf fall. Sometimes, adults damage shoots at the bud-break, but the second generation is usually the most economically damaging when remarkable pest densities affect grapes at the veraison. Moreover, the occurrence of many adults causes nuisance to pickers during harvest time. Specific economic thresholds have not been determined in the native areas, but those available for phylogenetically close species [7] can be considered as realistic.

In most cases, naturally occurring *E. vulnerata* antagonists, mainly mirid bugs (Heteroptera: Miridae) and mymarid wasps (Hymenoptera: Mymaridae) seemed unable to keep the pest population densities at acceptable levels for vine-growers [8]. The use of synthetic insecticides (e.g., neonicotinoids and pyrethroids) can achieve leafhopper control in conventional viticulture, but natural insecticides used in organic farms are less effective [9]. The side effects of both natural and synthetic insecticides are a matter of concern, and reduction of their use in Europe is the focus of the Directive 2009/128/EU [10] and, more recently, of the Farm to Fork Strategy [11]. Therefore, the identification of feasible alternatives to insecticides is a priority in Europe. Augmentative biocontrol strategies through the release of predators and parasitoids could be useful to control *E. vulnerata* populations [12,13,14].

In California vineyards, green lacewings (Neuroptera: Chrysopidae) were released to control leafhoppers, i.e., *Erythroneura variabilis* Beamer and *Erythroneura elegantula* Osborn, with positive results [15]. *Chrysoperla carnea* is a generalist predator that may prey upon more than 70 species belonging to five orders, but Homopterans represent the preferred targets [16]. It has been widely used in augmentative biological control tactics against aphids and lepidopterans [17,18]. *Orius majusculus* preys upon a variety of arthropod species, such as thrips, leafhoppers, aphids, lepidopterans, and spider mites [18,19,20]. It has been commonly detected in various crops (including grapevines) in North-eastern Italy, preying upon homopterans and spider mites [21,22]. Despite predation upon grape leafhoppers was observed in the latter studies, ad hoc experiments were not planned. Both species are well-known generalist predators in Italian vineyards [19,20,21,22,23,24] but their densities are often limited probably because of their susceptibility to pesticides [25].

Here we performed laboratory, semi-field and field experiments to evaluate the potential impact of *Chrysoperla carnea* Stephens (Neuroptera: Chrysopidae) and *Orius majusculus* (Reuter) (Hemiptera: Anthocoridae) releases for augmentative biocontrol of *E. vulnerata* in vineyards. We evaluated if the leafhopper constituted a prey for both generalist predators and assessed their prey consumption rate in laboratory experiments. Then we evaluated if both predators could reduce *E. vulnerata* populations in semi-field experiments on potted plants. Finally, the impact of both predators was assessed under a real use scenario in two field experiments.

## 2. Materials and Methods

In all the experiments we used *C. carnea* and *O. majusculus* provided by the biofarm Bioplanet (Cesena, Italy), which sells *C. carnea* as larvae and *O. majusculus* as adults and delivers the material in plastic bottles with netted caps. The bottles contain a mixture of inert material and predators. All the predators were used on the same day of their arrival, on the recommendation of the producer.

### 2.1. Laboratory Experiments

Laboratory experiments were carried out to assess the capacity of *C. carnea* and *O. majusculus* to prey upon *E. vulnerata* nymphs. Laboratory-reared 3rd instar nymphs of *E. vulnerata* were transferred onto grapevine leaf disks inside plastic Petri dishes (90 mm in diameter, 15 mm in height) used as experimental arenas. Grapevine leaves (cultivar Glera) were collected in the University of Padua (Italy) experimental farm and washed with water plus Tween (0.15% *w*/*w*) before the experiment. Three prey densities (5, 10 and 20 leafhopper nymphs per Petri dish) were considered as prey offer to predators. The experiment followed a full factorial design with 9 treatments defined by the combination of the factor prey density (i.e., 5, 10, 20 *E. vulnerata* nymphs/Petri dish) and the factor predator release (i.e., *C. carnea; O. majusculus;* Control), and each treatment comprised at least 4 replicates. Single *C. carnea* larvae or *O. majusculus* adults were transferred onto an experimental arena immediately after placing *E. vulnerata* nymphs. The number of living and preyed (showing clear signs of predation, i.e., with a completely emptied body) leafhopper nymphs were recorded after 24 h from the beginning of the experiment. Experimental arenas were maintained in a climatic chamber at 23 ± 2 °C and 70–80% relative humidity (RH) with a photoperiod of 16L:8D.

### 2.2. Semi-Field Experiments

Two semi-field trials were carried out using (about 35 cm tall) single potted vines that were confined inside insect-proof cages (W24.5 × D24.5 × H63.0 cm, with mesh of 680 µm, BugDorm-4S2260, MegaView Science Education Services Co., Ltd., Taichung, Taiwan). Cages were placed in outdoor conditions under the shade and protected from the rain. Experimental units were set up in the University of Padua’s experimental farm, and the experiments were carried out from May to August 2019 (T° average = 23.51 °C, min = 7.72 °C, max = 36.91 °C; RH average = 61.36%, min = 41.16%, max = 84.75%). *Erasmoneura vulnerata* infestation was set differently in the two experiments. In the first experiment, each vine was infested by 60 (2nd–4th instar) *E. vulnerata* nymphs. In the second experiment, three *E. vulnerata* adults (two females and one male) were confined to a vine and allowed to reproduce during June and July; then, adults were removed, and the number of living nymphs was estimated by using a portable magnifying lens before predator releases. Infestation density was assessed prior to predator releases. In both semi-field experiments three treatments were used: (1) *C. carnea* (3rd instar larvae) release; (2) *O. majusculus* (adults) release; (3) Control. Each treatment had five replicates and each vine was used only once. In field conditions, the producer recommends releasing a higher number of *C. carnea* larvae than *O. majusculus* adults (6–7 times more lacewings than pirate bugs) when controlling other homopteran pests. However, considering also results obtained in previous laboratory experiments and the specific conditions of semi-field studies (predators were confined into cages), we adopted the following release rate: 1 *C. carnea* larva/10 *E. vulnerata* nymphs and 1 *O. majusculus* adult/30 *E. vulnerata* nymphs. *Erasmoneura vulnerata* nymphs and the two predators were transferred into cages using a pencil brush. In the second experiment, *E. vulnerata* adults were confined using a mouth aspirator after being sexed, while a pencil brush was used for predators. In both experiments, *E. vulnerata* density was assessed two weeks after predator release by counting the number of living individuals inside each cage. The period considered for the evaluation was based on potted vine conditions. Longer time periods were not considered because plants in the control treatment were severely deteriorated and leaves started to fall.

For the first semi-field experiment, we calculated the survival rate for each treatment and for all cages as the ratio of the final number of nymphs over the initial number of nymphs. For the second experiment and all cages, we calculated the rate of change of *E. vulnerata* nymph density during the experiment using the following formula:

rt = Ln (Nt/Nt-τ)/τ.(1)

Nt-τ is the nymph density in each cage before predator releases, Nt is the number of nymphs in each cage at the end of the experiment, and τ is the time in days after predator release (i.e., 15 in both experiments). With rt > 0, indicate increase in nymph density, with rt < 0 means a decrease in nymph density, while rt = 0 means that the nymph density is stable.

At the conclusion of these experiments, the counting of the nymphs required pulling plants out of cages.

### 2.3. Field Experiments

The impact of *C. carnea* and *O. majusculus* on *E. vulnerata* populations was assessed in field conditions. Predators were released in two infested vineyards. The first trial was carried out in a vineyard located at Conegliano (North-eastern Italy, 45°52′53.05″ N, 12°17′00.26″ E, 77 m a.s.l.) in 2018. This vineyard comprised the cultivar Merlot and was trained with the Guyot system. The second trial was carried out in a vineyard located at Ponte di Piave (North-eastern Italy, 45°72′78.39″ N, 12°46′82.44″ E, 11 m a.s.l.) in 2019. It comprised the cultivar Cabernet Sauvignon, trained with the Bellussi system. No insecticides were applied in the selected vineyards during this study.

In the first experiment (2018), three treatments were compared: (1) *C. carnea* release; (2) *O. majusculus* release; (3) Control. Each treatment comprised four replicates, each having five vines (approximately 20 m^2^ of vine canopy). About 30 *C. carnea* larvae or 4 *O. majusculus* adults were released per m^2^ of canopy in the respective treatment. These figures were 50% higher than those recommended by the producer for the control of other homopteran pests on vegetables. We increased predator numbers because the permanent cordon of experimental vines was about at 1 m from the groundcover and this feature could favor the dispersal of released predators. Predators were released on 21 July. They were manually distributed on the permanent cordon and the canopy. Sampling was carried out to evaluate leafhopper and predator densities before (on 20 July) and after releases (on 28 July, 4 and 11 August). In each sampling date, 100 leaves per treatment were randomly collected and transferred to the laboratory, where they were observed under a Wild M3 stereomicroscope (10–60X) to assess the abundance of *E. vulnerata* and the released predators.

In the second experiment (2019), the same treatments considered in the previous experiment were compared. They comprised four replicates of three vines (approximately 24 m^2^ of canopy). As in the first experiment, predators were released using the same number of predators/m^2^ reported above but two releases were performed (on 9 and 21 August). Sampling was carried out to evaluate leafhopper and predator densities before and after releases. In each sampling date, 100 leaves per treatment were transferred to the laboratory to assess the abundance of *E. vulnerata* and the released predators using previous procedures.

In both experiments each plot was separated from the other plots by about 20 m.

### 2.4. Statistical Analysis

Data from laboratory experiments were analyzed with a generalized linear model using the GLIMMIX procedure of SAS ver. 9.4 [26] accounting for the binomial distribution of the data and using a logit link function. The number of surviving nymphs at the end of the experiment over the initial number was considered as the dependent variable. Prey density, predator release and their interaction were considered as independent variables and their effect was assessed with a χ^2^ test (*p* = 0.05). A post-hoc Tukey’s test (*p* = 0.05) on the least-square means was used for means separation.

A second analysis with a Generalized linear model (Poisson model with logarithmic link function) of data from laboratory experiment assessed if the number of preys consumed at different prey densities was different between the two predators. This analysis was performed with the proc GLIMMIX of SAS ver. 9.4 [26] and considering only the treatments with *C. carnea* and *O. majusculus*. Data for each nymph density was analyzed separately. The number of consumed prey was used as dependent variable. A *t*-test (*p* = 0.05) was used to assess the differences between the predators at different prey density.

In semi-field experiment (1) data were analyzed using a generalized linear model with a logit link function with the GLIMMIX procedure of SAS, ver. 9.4 [26]. The number of surviving nymphs at the end of the experiment over the initial numbers was considered as the dependent variable. A χ^2^ test (*p* = 0.05) was used to assess the effect of the independent variable, i.e., predator treatment. A Tukey’s test (*p* = 0.05) was used to evaluate differences among predators.

Data from the semi-field experiment (2) were analyzed with the general linear model within the MIXED procedure of SAS, ver. 9.4 [26]. Firstly, we assessed homogeneity of prey density among treatments prior to predator releases. Secondly, we analyzed the rate of prey numbers change. Differences among treatments were evaluated with an F test (*p* = 0.05). A post-hoc Tukey’s test (*p* = 0.05) was used for mean separation. The models’ assumptions were evaluated by inspecting diagnostic plots of model residuals and untransformed data were used.

A second analysis of data from semi-field experiment (2) evaluated the presence of differences between the percentage of 1st–2nd instar leafhopper nymphs over the total number of nymphs in each treatment at the end of the experiment. This analysis was performed using a generalized linear model with a logit link function with the proc GLIMMIX of SAS, ver. 9.4 [26]. The number of 1st–2nd instar nymphs over the total number of nymphs was used as dependent variable. A χ^2^ test was used to assess the effect of treatments followed by a Tukey’s test (*p* = 0.05) for treatment mean separation.

Data from field trials was analyzed using a repeated measures linear mixed Model with the MIXED procedure of SAS, ver. 9.4 [26]. The model was used to test the effect of predator releases, time of sampling, and their interactions on the number of *E. vulnerata* motile forms observed during the experiment. The effect of predator release, time of sampling and their interaction was tested using an F test (*p* = 0.05). Degrees of freedom were estimated with the Kenward and Roger method. A Tukey’s test (*p* = 0.05) to the least-square means was applied to evaluate the differences among treatments. The models’ assumptions were evaluated by inspecting diagnostic plots of model residuals, and data on leafhoppers density were log (*n* + 1) transformed before the analysis. Differences among treatments before predator release were verified with the MIXED procedure of SAS, ver. 9.4 [26] and evaluated with an F test (*p* = 0.05).

## 3. Results

### 3.1. Laboratory Experiment

**Figure 1:**
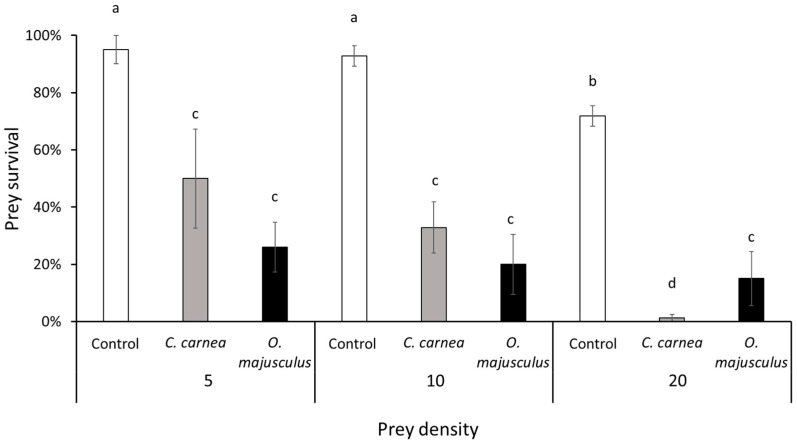
Survival rates (mean ± standard error (SE)) of *Erasmoneura vulnerata* nymphs offered as prey to *Chrysoperla carnea* or *Orius majusculus* in the laboratory. Different letters indicate significant differences at Tukey’s test (*p* = 0.05).

All predators survived during the laboratory experiment. *Chrysoperla carnea* and *O. majusculus* actively preyed upon *E. vulnerata* nymphs. Nymph survival was influenced by predator release, initial nymph density and their interaction (respectively: χ^2^ = 114.06; d.f. = 2; *p* < 0.0001; χ^2^ = 27.48; d.f. = 2; *p* < 0.0001; χ^2^ = 12.47; d.f. = 2; *p* = 0.0141). Although prey survival appeared to decline with initial density for both predator species the lowest survival rate was observed on *C. carnea* treatment where 20 nymphs were offered to the predator (Figure 1).

**Figure 2:**
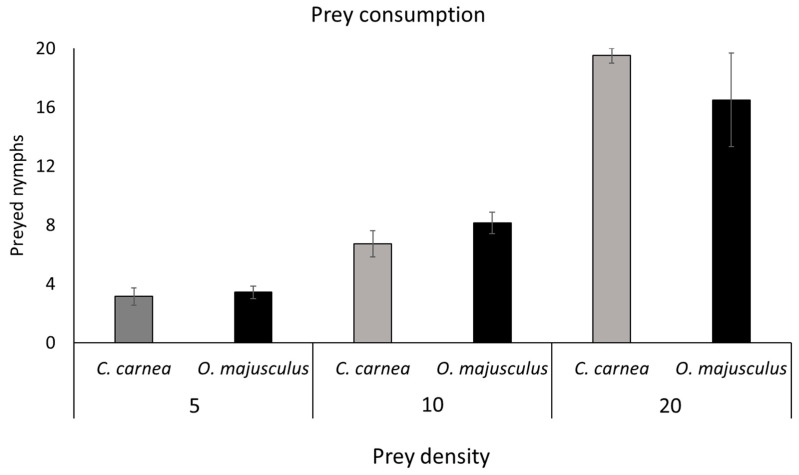
Number of prey consumed by *Chrysoperla carnea* and *Orius majusculus* at different prey densities in the laboratory.

No significant differences in prey consumption were found among predators at the different prey densities (**5**: t = 0.29; d.f. = 12; *p* = 0.773; **10**: t = 0.98; d.f. = 12; *p* = 0.346; **20**: t = 1.00; d.f. = 6; *p* = 0.356; Figure 2).

### 3.2. Semi-Field Experiments

#### 3.2.1. Semi-Field Experiment 1

**Figure 3:**
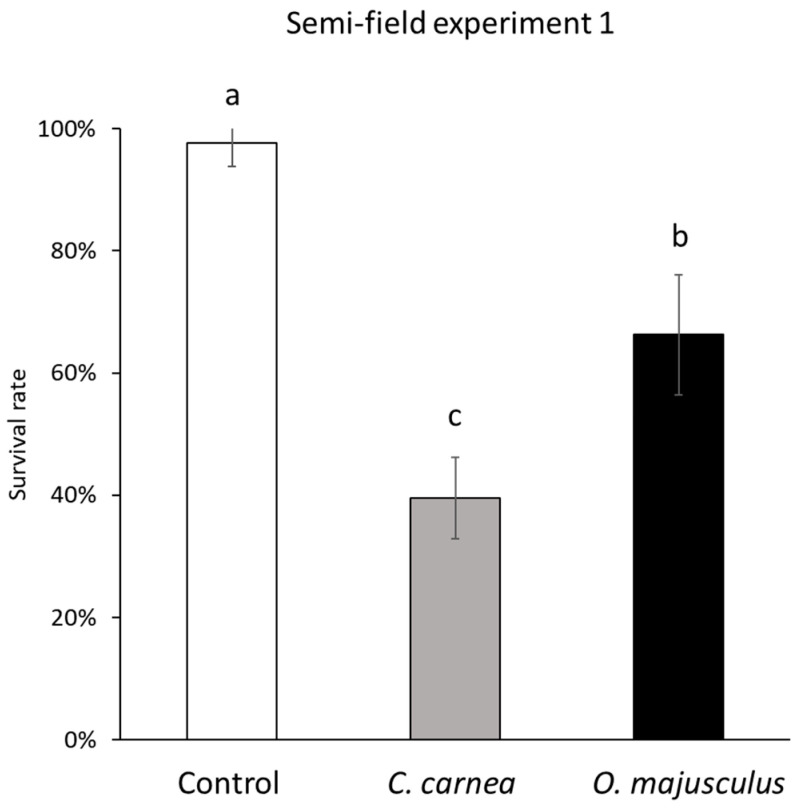
Survival rates (mean ± SE) of *Erasmoneura vulnerata* nymphs observed in the three treatments (*Chrysoperla carnea*, *Orius majusculus*, and Control) under comparison in the semi-field experiment (1), two weeks after predator releases. In this experiment, 60 *E. vulnerata* nymphs per cage were introduced at the beginning of the trial. Predators were released in a ratio of 1/10 *E. vulnerata* nymphs (*C. carnea*) or 1/30 *E. vulnerata* nymphs (*O. majusculus*). Different letters indicate significant differences in the Tukey’s test (*p* = 0.05).

In the first experiment, predator releases significantly reduced leafhopper survival χ^2^ = 178.36; d.f. = 2, 12; *p* < 0.0001; Figure 3). Different outcomes between the two predatory species were recorded, as *E. vulnerata* decreased more in *C. carnea* compared to *O. majusculus* treatment (χ^2^ = 5.89; d.f. = 1, 12; *p* < 0.0001; Figure 3).

#### 3.2.2. Semi-Field Experiment 2

**Figure 4:**
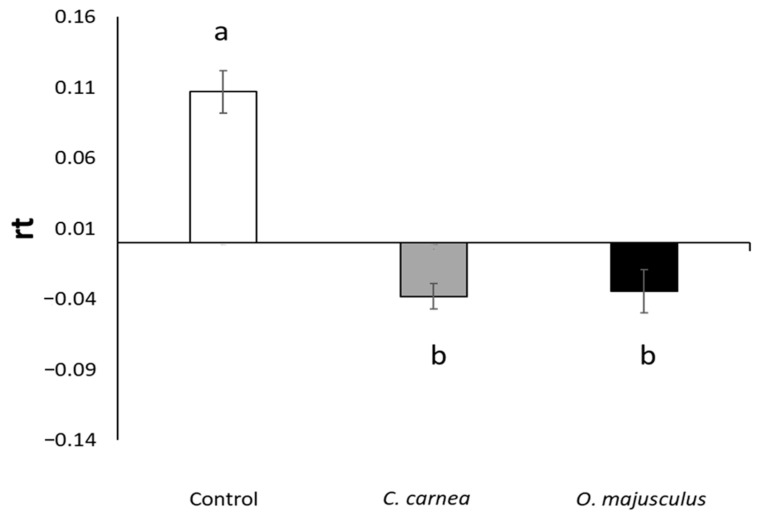
Rate of change of *Erasmoneura vulnerata* nymph density (mean ± SE) observed in semi-field experiment (2) in the three treatments (*Chrysoperla carnea*, *Orius majusculus*, and Control), two weeks after predator releases. The initial nymph density (about 70 nymphs per cage) was generated by the inoculation of two adult females. Predators were introduced in a ratio of 1/10 *E. vulnerata* nymphs (*C. carnea*) or 1/30 *E. vulnerata* nymphs (*O. majusculus*). Data are expressed as rate of change of nymph numbers due to predator introduction. Different letters indicate significant differences at Tukey’s test (*p* = 0.05).

In the second experiment, the number of *E. vulnerata* nymphs detected prior to the predator releases was similar among the cages (70 ± 5.04 mean ± standard error (SE); F = 0.67; d.f. = 2, 12; *p* = 0.528). Leafhopper densities were significantly reduced by the two predators (F= 37.88; d.f. = 2, 12; *p* < 0.0001; Figure 4), and no differences between them were observed (F = 0.19; d.f. = 1, 12; *p* = 0.853; Figure 4).

**Figure 5:**
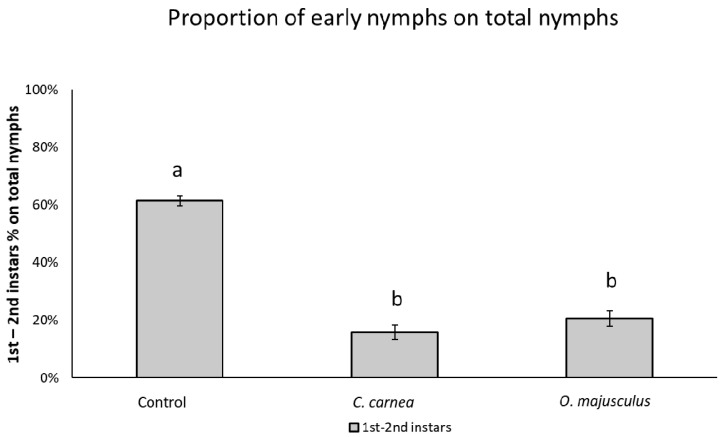
Proportion of *Erasmoneura vulnerata* 1st–2nd instar nymphs over the total number of nymphs observed at the end of the semi-field experiment (2), in the three treatments (*Chrysoperla carnea*, *Orius majusculus*, and Control) two weeks after predator releases. The percentage (mean ± SE) was calculated as 1st–2nd instar nymphs over of the final number of nymphs. Different letters indicate significant differences at Tukey’s test (*p* = 0.05).

Concerning the nymph numbers at the end of the experiment, a higher proportion of 1st–2nd instar nymphs were observed in the control plots compared to the other two treatments (χ^2^ = 86.22; d.f. = 2, 12; *p* < 0.0001; Figure 5).

### 3.3. Field Experiments

**Figure 6:**
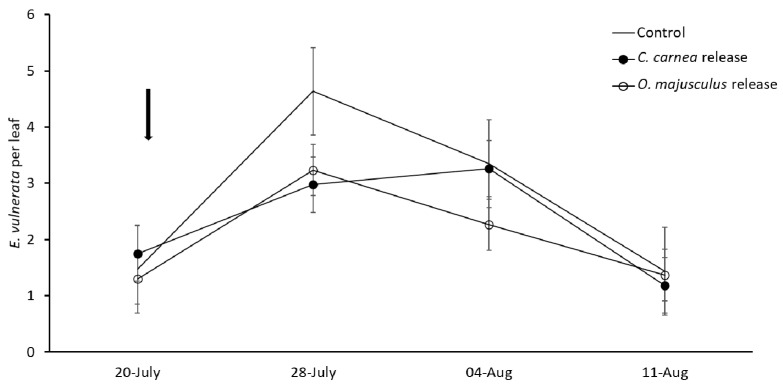
*Erasmoneura vulnerata* densities (mean ± SE) in vineyard plots characterized or not by predator releases (experiment 1, 2018). The arrow indicates the predator release event.

In the first experiment (2018, Figure 6), *E. vulnerata* densities slightly increased after predator releases and then declined in all treatments. Leafhopper populations significantly fluctuated over sampling dates (F = 36.29; d.f. = 3, 27; *p* < 0.0001). Predator releases significantly affected leafhopper population densities (F = 4.03; d.f. = 16.6; *p* = 0.037; Figure 6) with different outcomes between the two predators. Leafhopper densities in *O. majusculus* release plots were lower as compared to the control plots (F = 2.82; d.f. = 16.6; *p* = 0.036) while no differences emerged between *C. carnea* release plots and the control (F = 1.66; d.f. = 2, 16.6; *p* = 0.348). No significant differences in leafhopper densities were found in *O. majusculus* and *C. carnea* release plots (F = 1.17; d.f. = 1, 16.6; *p* = 0.78). The occurrence of both predators in leaf samples was low (max. 0.04/leaf) and no differences were detected among treatments (treatment: F= 2.36; d.f. = 2, 29.3; *p* = 0.09; time: F= 2.42; d.f. = 2, 15.9; *p* = 0.1208, treatment*time: F= 2.36; d.f. = 6, 29.6; *p* = 0.056; data not shown).

**Figure 7:**
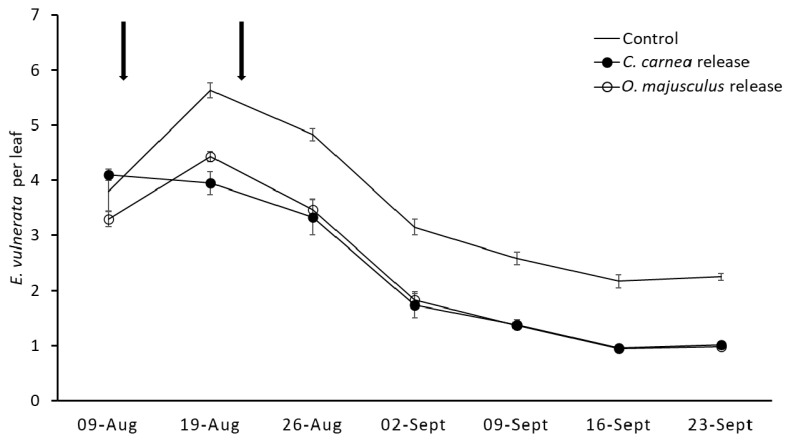
*Erasmoneura vulnerata* densities (mean ± SE) in vineyard plots characterized or not by predator releases (experiment 2, 2019). Each arrow indicates predator release events.

In the second experiment (2019, Figure 7) *E. vulnerata* densities slightly increased in the second sampling date and then declined in all treatments. Predator releases significantly affected leafhopper population densities compared to the control (F = 136.9; d.f. = 2, 71; *p* < 0.0001; Figure 7). The effect of time and the interaction “treatment*time” were significant (F = 453.81; d.f. = 7, 71; *p* < 0.0001; F = 10.04; d.f. = 14, 71; *p* < 0.0001, respectively). No differences were observed prior to predator release, while afterward lower densities were observed in the two predator release plots compared to the control plots (*C. carnea*: F = 14.03; d.f. = 1, 71; *p* < 0.0001; *O. majusculus*: F = 14.71; d.f. = 1, 71; *p* < 0.0001; Figure 7). No differences in the density of *E. vulnerata* emerged between the two predators (F = 0.69; d.f. = 1, 71; *p* = 0.494). The occurrence of both predators in leaf samples was low (max. 0-04/leaf) and thus there were no differences in their numbers; (treatment: F = 2.26; d.f. = 2, 71; *p* = 0.111; time: F = 0.44; d.f. = 7, 71; *p* = 0.8718, treatment*time: F = 0.65; d.f. = 14, 71; *p* = 0.815).

## 4. Discussion

Both predators preyed actively upon *E. vulnerata* nymphs and significantly reduced their densities compared with the control in all experiments. In the laboratory experiments up to 20 nymphs of *Erasmoneura vulnerata* were preyed upon by a single *C. carnea* larva in one day, while an adult of *O. majusculus* consumed up to 16 preys in the same time period. Both predators seemed to increase the prey consumption with increasing *E. vulnerata* prey offer density. In our experiment, we used a limited range of prey density which did not allow us to delineate a functional response that could help to describe predator–prey relationships [27,28]. However, the primary purpose of our laboratory experiments was to investigate whether the leafhopper *E. vulnerata* constitutes a suitable prey for the two predators, as an initial step in their evaluation as biological control agents [29,30].

Interactions between *O. majusculus* and leafhoppers were investigated in Spain. Ardanuy [16] observed early-season increases of *Orius* spp. in maize fields potentially related to the occurrence of leafhoppers, in particular *Zyginidia scutellaris* (Herrich-Schäffer). Predators were markedly attracted by volatiles emitted from maize plants infested with *Z. scutellaris*. Feeding by *Z. scutellaris* induces the emission of maize’s HIPVs (herbivore-induced plant volatiles) that attract anthocorids into maize fields. Few studies have examined the predation by *C. carnea* upon leafhoppers. Erlandson and Obrycki [31] compared the predation activity of *C. carnea* on the leafhopper *Empoasca fabae* Harris with that exhibited by the anthocorid *Orius insidiosus* (Say) and the coccinellid *Coleomegilla maculata* (De Geer). *Chrysoperla carnea* appeared to be the most voracious among tested predators, especially in the high-prey density trials [31]. The impact of lacewings on grape leafhoppers has been evaluated in California, where Daane et al. [15] released *C. carnea* in vineyards infested by *E. variabilis* and *E. elegantula*. In their first trial, *C. carnea* larvae were released into cages and leafhopper densities were reduced by 23.5–30.3%. In our first semi-field experiment, lacewing larvae seemed to perform better with more than double reduction compared to that obtained by Daane et al. [15].

Previous studies with the release of *C. carnea* in vineyards for the control of leafhopper were achieved by the release of *C. carnea* larvae in vineyards where a significant reduction in leafhopper density was obtained (33.6% and 31.4% in the first and second generations, respectively), with about 20,000 larvae released per hectare [15]. However, unsatisfactory results were obtained in other trials using the same approach [15]. Differences in release methods and prey densities were claimed as possible factors affecting these contrasting results. Furthermore, prey densities had a significant role in the outcome of *C. carnea* releases, as predators could not reduce leafhopper densities below the economic injury level in high pest pressure conditions [15], even if in our laboratory experiment the highest daily prey consumption was observed at the highest leafhopper density. Aspects related to augmentative releases of green lacewings (including *C. carnea*) were further evaluated in California vineyards [7]. A mixture of lacewing eggs and corn grit placed in paper cups was distributed to every 5th vine in every other row; this system was associated with low egg hatching and larvae dispersal. Egg hatching increased when they were dropped onto the vines from a moving flatbed trailer. In other trials, the effect of increasing release rates (from 6175 to 1,235,000 eggs or larvae per hectare) was compared, but prey numbers were not correlated with release densities. Releases were more effective when nymphs were at the beginning of the generation (before peak); these findings are in accordance with what we found in the second semi-field experiment (2), where a higher impact of the predators on the youngest stages of the leafhoppers (1st–2nd instars) was evident when observing the final composition of the nymphs. The 1st–2nd instar nymphs are easier prey to catch and require less energy consumption to prey upon. Anyway, in Daane et al. [7] experiments, larval releases are confirmed to be more effective than egg releases.

In our field studies, we reported a 30% decrease in *E. vulnerata* abundance compared to the control plots, similar to findings obtained by Daane et al. [15], in California, on phylogenetically close leafhopper species. However, the results of our field experiments (for both predator species) were less convincing compared to those reported in the laboratory and semi-field studies. These discrepancies could be due to many factors, and among them, release techniques, the occurrence of alternative prey, and climatic conditions could be the most important [32]. In grapevine training systems we considered, the permanent cordon grows at 1–2 m from the ground level. Therefore, several released predators could fall and disperse after releases. Predators’ dispersion and cannibalism may be reduced using cups fastened on the cordon, but it is a time-consuming activity that can increase the total costs. In the 2019 field experiment, we increased the number of released predators to overcome this issue and to try to increase the predator impact, but results were not fully satisfactory. In the experimental vineyard used in 2019, spider mites occurred at a moderate level in all the treatments. Spider mites may be an alternative prey to *E. vulnerata* nymphs for the tested predators. For instance, *C. carnea* resulted in being an effective predator of spider mites in many studies [33,34,35]. On the other hand, previous research performed in order to assess prey preferences of *Orius* spp., comparing spider mites and other important pests, showed that mites were not the favorite prey [36,37,38]. The drought typical of summertime in the study area could also have affected release success. Furthermore, it is important to consider predator mobility. Predator movements often follow the prey density in these environments [39], and it is reasonable that *E. vulnerata* predators could have moved to control plots when prey starts to decline in the release plots. Unfortunately, a low number of released predators was detected in leaf samples, probably due to their high mobility.

Nevertheless, the present study provides perspectives for the development of an augmentative biocontrol program against *E. vulnerata* in vineyards. This could be of increasing interest in the context of European viticulture, where the use of pesticides is going to be increasingly restricted. Based on our results, obtained in laboratory, semi-field, and field experiments, we can conclude that both predators can potentially be implemented in biocontrol programs. The second field experiment results are auspicious and highlighted how both predators performed to control the invasive pests. Future studies are needed to elucidate the different factors that may influence these generalist predators’ effectiveness in controlling *E. vulnerata* populations in realistic conditions, particularly multi-trophic interactions with naturally occurring predators and alternative prey. At the same time, experiments are required to define the economic thresholds for *E. vulnerata* and the best timing for the release of these predators in order to point out appropriate release programs [9,40].

## Data Availability

The data presented in this study are available on request from the corresponding author. The data are not publicly available due to privacy.

## References

[1] Zimmerman R., Kondratieff B., Nelson E., Sclar C. (1996). The life history of two species of grape leafhoppers on wine grapes in western Colorado. J. Kansas Entomol. Soc..

[2] Duso C., Bressan A., Mazzon L., Girolami V. (2005). First record of the grape leafhopper *Erythroneura vulnerata* Fitch (Hom., Cicadellidae) in Europe. J. Appl. Entomol..

[3] Duso C., Zanettin G., Gherardo P., Pasqualotto G., Raniero D., Rossetto F., Tirello P., Pozzebon A. (2020). Colonization patterns, phenology and seasonal abundance of the nearctic leafhopper *Erasmoneura vulnerata* (Fitch), a new pest in european vineyards. Insects.

[4] Seljak G. (2011). First record of the Nearctic leafhopper *Erasmoneura vulnerata* (Fitch, 1851) [Hemiptera, Cicadomorpha: Cicadellidae] in Slovenia. Acta Entomol. Slov..

[5] Rizzoli A., Battelli R., Conedera M., Jermini M. (2020). First record of *Erasmoneura vulnerata* Fitch, 1851 (Hemiptera, Cicadellidae, Typhlocybinae) in Switzerland. Alp. Entomol..

[6] Duso C., Moret R., Manera A., Berto D., Fornasiero D., Marchegiani G., Pozzebon A. (2019). Investigations on the grape leafhopper *Erasmoneura vulnerata* in North-eastern Italy. Insects.

[7] Daane K.M., Rosenheim J.A., Smith R.J., Coviello R.L., Bettiga L.J. (2013). Western Grape Leafhopper. Grape Pest Management.

[8] Duso C. (2019). Personal communication.

[9] Tirello P., Marchesini E., Gherardo P., Raniero D., Rossetto F., Pozzebon A., Duso C. (2021). The Control of the American Leafhopper *Erasmoneura vulnerata* (Fitch) in European Vineyards: Impact of Synthetic and Natural Insecticides. Insects.

[10] (2009). EU Directive 2009/128/EU of the European Parliament and the Council of 21 October 2009 Establishing a Framework for Community Action to Achieve the Suistanable Use of Pesticides.

[11] Farm to Fork Strategy. A Farm to Fork Strategy for a Fair, Healthy and Environmentally-Friendly Food System. Communication from the Commission to the European Parliament, the Council, the European Economic and Social Committee and the Committee of the Regions; COM/2020/381 Final. https://ec.europa.eu/food/sites/food/files/safety/docs/f2f_actionplan_2020_strategy-info_en.pdf.

[12] Parella M.P., Heinz K.M., Nunney L. (1992). Biological control through augmentative releases of natural enemies: A strategy whose time has come. Am. Entomol..

[13] Symondson W.O.C., Sunderland K.D., Greenstone M.H. (2002). Can generalist predators be effective biocontrol agents?. Annu. Rev. Entomol..

[14] Plouvier N.W., Wajnberg E. (2018). Improving the efficiency of augmentative biological control with arthropod natural enemies: A modeling approach. Biol. Control.

[15] Daane K.M., Yokota G.Y., Zheng Y., Hagen K.S. (1996). Inundative release of common green lacewings (Neuroptera: Chrysopidae) to suppress *Erythroneura variabilis* and *E. elegantula* (Homoptera: Cicadellidae) in vineyards. Environ. Entomol..

[16] Ardanuy A., Albajes R., Turlings T.C.J. (2016). Innate and learned prey-searching behavior in a generalist predator. J. Chem. Ecol..

[17] Hagen K.S., Slansky F., Rodriguez J.G. (1987). Nutritional ecology of terrestrial insect predators. Nutritional Ecology of Insects, Mites, Spiders, and Related Invertebrates.

[18] New T.R. (1975). The biology of Chrysopidae and Hemerobiidae (Neuroptera), with reference to their usage as biocontrol agents: A review. Trans. R. Entomol. Soc. Lond..

[19] Bosco L., Tavella L. (2013). Distribution and abundance of species of the genus Orius in horticultural ecosystems of northwestern Italy. Bull. Insectol..

[20] Alvarado P., Baltà O., Alomar O. (1997). Efficiency of four heteroptera as predators of *Aphis gossypii* and *Macrosiphum euphorbiae* (Hom.: Aphididae). Entomophaga.

[21] Montserrat M., Albajes R., Castane C. (2000). Functional response of four Heteropteran predators preying on greenhouse whitefly (Homoptera: Aleyrodidae) and western flower thrips (Thysanoptera: Thripidae). Environ. Entomol..

[22] Duso C., Girolami V. (1983). Ruolo degli Antocoridi nel controllo del *Panonychus ulmi* Koch nei vigneti. Boll. Ent. Bol..

[23] Paoletti M.G. (1984). La vegetazione spontanea dell’agroecosistema e il controllo dei fitofagi del mais. Giorn. Fitopatol..

[24] Girolami V. (1987). Mites of vineyards and control strategies. Proceedings of the Meeting EC Experts’ Group “Integrated Pest Control in Viticulture”, Portoferraio, Italy, 26–28 September 1985.

[25] Sterk G., Hassan S.A., Baillod M., Bakker F., Bigler F., Blümel S., Bogenschütz H., Boller E., Bromand B., Brun J. (1999). Results of the seventh joint pesticide testing programme carried out by the IOBC/WPRS Working Group “Pesticides and Beneficial Organisms”. BioControl.

[26] (2016). SAS PROC User’s Manual.

[27] Holling C.S. (1966). The functional response of invertebrate predators to prey density. Mem. Entomol. Soc. Can..

[28] Juliano S.A. (2001). Nonlinear curve fitting: Predation and functional response curves. Design and Analysis of Ecological Experiments.

[29] Waage J.K., Mackauer M., Ehler L.E., Roland J. (1990). Ecological theory and the selection of biological control agents. Critical Issues in Biological Control..

[30] Lester P.J., Harmsen R. (2002). Functional and numerical responses do not always indicate the most effective predator for biological control: An analysis of two predators in a two-prey system. J. Appl. Ecol..

[31] Erlandson L.A.W., Obrycki J.J. (2010). Predation of immature and adult *Empoasca fabae* (Harris) (Hemiptera: Cicadellidae) by three species of predatory insects. J. Kansas Entomol. Soc..

[32] Collier T., Van Steenwyk R. (2004). A critical evaluation of augmentative biological control. Biol. Control.

[33] Hassanpour M., Nouri-Ganbalani G., Mohaghegh J., Enkegaard A. (2009). Functional response of different larval instars of the green lacewing, *Chrysoperla carnea* (Neuroptera: Chrysopidae), to the two-spotted spider mite, *Tetranychus urticae* (Acari: Tetranychidae). J. Food Agric. Environ..

[34] Mena Y.M., Mesa N.C., Escobar A., Pérez S. (2020). Evaluation of Phytoseiidae mites and *Chrysoperla carnea* (Stephens) on the control of *Tetranychus urticae* in *Carica papaya* L. Agron. Colomb..

[35] Khan A.A., Zaki F.A. (2008). Predatory response of *Chrysoperla carnea* (Stephens) (Neuroptera: Chrysopidae) feeding on the Euonymus aphid, *Aphis fabae* solanella Theobald (Homoptera: Aphelinidae) in Kashmir. J. Biol. Control..

[36] Wearing C.H., Colhoun K. (1999). Development of *Orius vicinus* (Ribaut) (Heteroptera: Anthocoridae) on different prey. Biocontrol Sci. Technol..

[37] Xu X., Enkegaard A. (2009). Prey preference of *Orius sauteri* between Western Flower Thrips and spider mites. Entomol. Exp. Appl..

[38] Blaeser P., Sengonca C., Zegula T. (2004). The potential use of different predatory bug species in the biological control of Frankliniella occidentalis (Pergande) (Thysanoptera: Thripidae). J. Pest Sci..

[39] Ives A., Kareiva P., Perry R. (1993). Response of a Predator to Variation in Prey Density at Three Hierarchical Scales Lady Beetles Feeding on Aphids. Ecology.

[40] Rossetto F. (2019). Effetti di insetticidi su *Erasmoneura vulnerata* e valutazioni sui danni causati dall’insetto. Second-cycle degree. Agricultural Science and Technology.

